# Identification of GPX3 and JUN as Tumor Suppressors in Thyroid Cancer through Integrated WGCNA and Mendelian Randomization

**DOI:** 10.7150/jca.104687

**Published:** 2025-02-18

**Authors:** Renjie Zhang, Yutao Chen, Sha Xu, Xue Gu, Hui Ye

**Affiliations:** 1 Department of Thyroid Surgery, Affiliated Hospital of Guizhou Medical University, Guiyang, China.; 2 Department of Oncology, Guizhou Medical University Affiliated Cancer Hospital, Guiyang, China.

**Keywords:** thyroid cancer, GPX3, JUN, immunity, proliferation, migration

## Abstract

**Background:** Thyroid cancer (TC) ranks among the most common malignancies globally, with an increasing incidence among younger populations. While papillary thyroid carcinoma (PTC) generally has a favorable prognosis, other forms of TC, such as anaplastic thyroid carcinoma (ATC), are associated with poor outcomes. Although specific mutations, such as BRAF^V600E^, have been identified in certain types of TC, the underlying mechanisms remain largely unclear. Therefore, there is a critical need to further explore therapeutic targets associated with malignant tumors to improve treatment outcomes.

**Method:** We integrated eQTL data from European populations with RNA-Seq data from TC patients obtained from TCGA and multiple GEO databases. Through differential expression analysis, WGCNA, and Mendelian randomization (MR) analysis, we sought to identify potential gene therapy targets in TC. Additionally, we explored the biological behaviors of these targets using various cellular biology assays, such as MTT, colony formation, wound healing, and Transwell assays. Molecular biology techniques, including Western blot, were employed to investigate the underlying mechanisms.

**Result:** Differential expression analysis across six GEO datasets identified 649 genes associated with TC. Subsequent WGCNA analysis of the GSE6339 dataset revealed 2,739 genes, and MR analysis further identified 189 genes. The intersection of these datasets highlighted four key genes: TIAM1, RAP1GAP, GPX3, and JUN. GO analysis linked these genes to "response to oxidative stress" and "regulation of GTPase activity". KEGG pathway analysis demonstrated significant enrichment in pathways including "Glutathione metabolism", "cAMP signaling pathway", "Rap1 signaling pathway", "Tight junction", and "Thyroid hormone synthesis". Further, single-gene GSEA analyses suggested distinct pathways through which each gene may influence TC progression. Immune profiling revealed marked differences in immune cell populations, notably CD8+ T cells, monocytic lineage cells, neutrophils, NK cells, and T cells, between normal and cancerous thyroid tissues. Notably, RAP1GAP, GPX3, and JUN were implicated in the regulation of Treg and follicular helper T cell functions. The differential expression of these genes was rigorously validated using TCGA dataset and six additional GEO datasets. While the tumor-suppressive roles of TIAM1 and RAP1GAP have been previously established, our findings reveal that the overexpression of GPX3 and JUN significantly impairs the proliferative and migratory capacities of TC cells, underscoring their potential as therapeutic targets.

**Conclusion:** This study identifies GPX3 and JUN as critical tumor suppressor genes in TC, with their function closely linked to T regulatory cells and follicular helper T cells. The overexpression of GPX3 and JUN demonstrates significant tumor-suppressive activity, highlighting their potential as effective therapeutic targets in combating TC.

## Introduction

According to the Global Cancer Statistics 2020, TC is the ninth most common malignancy worldwide, with its incidence continuing to rise 1. Approximately 44,000 individuals are diagnosed with TC in the United States each year 2. Differentiated thyroid cancer (DTC) is the most prevalent subtype, and the current treatment strategies include surgery, selective radioactive iodine (RAI) therapy, and thyroid-stimulating hormone (TSH) suppression 3. Although these treatments are effective for most patients, anaplastic thyroid cancer (ATC) remains extremely aggressive and one of the deadliest malignancies, with cancer-specific mortality rates ranging from 98% to 99% 4. Additionally, TC is the most frequently diagnosed cancer in people between the ages of 15 and 29 1. Given these challenges, there is an urgent need to understand the mechanisms underlying TC development and progression to facilitate the development of novel early diagnostic tools, monitoring strategies, and targeted therapies.

GPX3 encodes a protein that is part of the glutathione peroxidase family, which plays a role in protecting cells from oxidative damage 5. Recent studies have revealed that promoter hypermethylation leads to the downregulation of GPX3 expression in a variety of human malignancies, such as bladder cancer 6. lung cancer 7, and hepatocellular carcinoma 8. However, the precise mechanisms remain unclear. In ovarian cancer, GPX3 has been reported to support cancer progression by modulating the extracellular redox environment 9. GPX3, in prostate cancer, this gene is either deleted or methylated, leading to the suppression of tumor growth and metastasis 10. Despite these findings, the biological role of GPX3 in TC has yet to be fully explored.

JUN is a gene that encodes a protein associated with diseases such as various malignant tumors 11-14. the AP-1/c-Jun~Fra-2 dimer drives c-Myc-induced hepatocarcinogenesis 13. JUN is involved in pathways like Toll-like receptor 3 (TLR3) signaling and prolactin signaling and plays a role in transcriptional activation of USP28 in colorectal cancer 15. However, the potential functions of JUN in TC have not yet been explored.

Mendelian randomization (MR) is a statistical method that uses genetic variants as instrumental variables to assess causal relationships between exposures and disease outcomes 16. Since genetic variants are randomly assigned at conception, they are generally independent of environmental and other confounding factors 17. This reduces the influence of confounders and allows for a clearer understanding of potential causal relationships, rather than mere associations 18. WGCNA (Weighted Gene Co-expression Network Analysis) is a powerful tool used to construct gene co-expression networks and identify modules of highly correlated genes. By applying various biological methodologies, we can more accurately and comprehensively investigate the factors involved in TC, providing valuable insights for the identification of precise gene targets for future therapeutic interventions.

## Materials and Methods

### Study design

**Figure 1:**
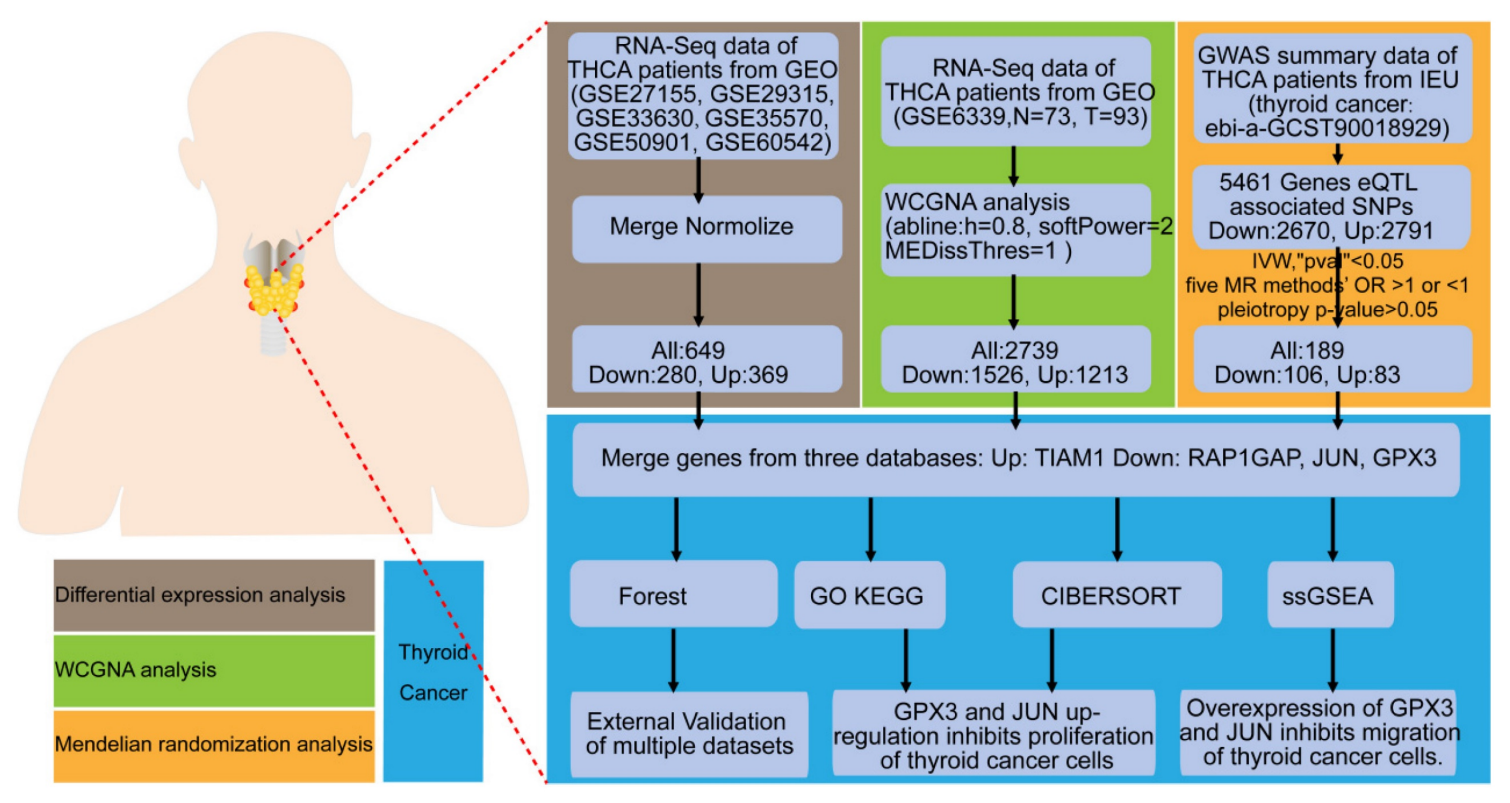
** Flow chart of the study.** Identification and experimental validation of genes associated with the occurrence and progression of TC using differential expression analysis, WGCNA, and MR approaches.

This study is a comprehensive bioinformatics analysis complemented by experimental validation in cellular models. The primary objective is to evaluate multiple gene expression datasets and identify biomarkers associated with TC. The general workflow is illustrated in the Figure 1.

### Data sources and preprocessing

We obtained seven TC datasets (GSE6339 19, GSE27155 20, 21, GSE33630 22, 23, GSE35570 24, GSE50901 25, 26, GSE60542 27) from the Gene Expression Omnibus (GEO) database as the training set, and selected six additional datasets (GSE3467 28, GSE3678 29, GSE9115 30, GSE65144 31, GSE104005 32, GSE129562 33) as the validation set. Detailed information regarding these datasets can be found in Supplementary Table 1. Inclusion Criteria: Patients who have been pathologically diagnosed with thyroid cancer; Patients must have complete genomic data available; participants must provide written informed consent to participate in the study and to allow the use of their data for analysis. Exclusion Criteria: Patients who are clinically unassessable; Genomic data that are incomplete, missing, or duplicated. Initially, we utilized the 'Combat' function in the "sva" package in R to eliminate batch effects., and combined the six datasets using the "sva" function. Additionally, RNA-Seq data from normal individuals and tumor patients (TCGA-THCA) obtained from The Cancer Genome Atlas (TCGA) were included as a validation set. The clinical characteristics of thyroid cancer, derived from the TCGA database, are presented in Supplementary Table 2. All the data were log_2_-transformed and missing values were excluded.

### Identification of DEGs between normal and tumor tissues in TC

We employed the "limma" package in R to identify differentially expressed genes (DEGs) in the GEO expression datasets, using |log_2_FC(fold change)| > 0.585 and adjusted *p*-value < 0.05. Next, WGCNA was conducted on the GSE6339 dataset, which is a method for data reduction and unsupervised classification. The co-expression network was constructed using the 'WGCNA' package in R with the merged matrix expression profiles. The parameters for network construction were set as follows: MEDissThres = 0.25, minModuleSize = 60, corType = "Pearson". Key genes associated with clinical traits were identified within each co-expression module. Following gene clustering, a heatmap was generated to illustrate the correlation between modules and phenotypes.

For MR analysis, expression quantitative trait loci (eQTL) data were obtained from the IEU OpenGWAS project (*https://gwas.mrcieu.ac.uk/*), which contains 19,942 genes. MR was conducted using the "TwoSampleMR" package in R, with conditions set as follows: *p*1 = 5e-08, *p*2 = 5e-08, clump = T, kb = 10000, R^2^ = 0.001. We further calculated R^2^ and *F*-statistics, filtering data based on an *F*-value > 10 to remove weak instruments. Outcome data for TC were retrieved from the GWAS summary database (Thyroid Cancer GWAS ID: ebi-a-GCST90018929) in the IEU OpenGWAS project, which includes 1,054 TC cases and 490,920 controls, encompassing 24,198,226 SNPs 34. Mendelian randomization analysis was performed, followed by heterogeneity analysis, pleiotropy tests, and leave-one-out analysis. Genes whose eQTLs were considered potential DEGs if they met the following criteria: consistent odds ratio (OR) direction across five methods, IVW *p*-value < 0.05, and pleiotropy *p*-value > 0.05. Genes that were consistently upregulated or downregulated across the three methods of differential expression analysis, WGCNA, and MR analysis were considered as DEGs.

### GO and KEGG analysis

The "clusterProfiler" package was utilized for Gene Ontology (GO) and KEGG analysis, which were considered statistically significant with a *p*-value < 0.05 and a false discovery rate (FDR) < 0.05.

### Gene Set Enrichment Analysis (GSEA)

GSEA was employed to estimate pathway alterations and cancer-related changes in gene expression datasets. This computational method was applied to GEO-TC merge patient gene expression data, where samples were grouped into high and low expression groups based on the median expression values. GSEA was performed using the gene sets "c5.go.symbols.gmt" and "c2.cp.kegg.Hs.symbols.gmt".

### Immune analysis

The CIBERSORT algorithm was applied to estimate the infiltration levels of 22 immune cell types based on the gene expression profiles of GEO-TC merge patients. Only results with a *p*-value < 0.05 were considered significant, and the infiltration levels were visualized using bar plots and boxplots. Correlations between the expression levels of the four genes and immune cell infiltration were assessed using R software.

### Tumor microenvironment analysis

The tumor samples were scored using the "estimate" package in R to obtain StromalScore, ImmuneScore, ESTIMATEScore, and TumorPurity values, followed by a differential analysis of the tumor microenvironment between normal and tumor samples. Immune cell infiltration was further analyzed using the "MCPcounter" R package, and heatmaps were generated to visualize differences in immune cell infiltration between normal and tumor tissues.

### Cell lines

The human TC cell lines CAL-62 and KHM-5M were obtained from Lisang Bio (Chongqing, China). CAL-62 cells were cultured in DMEM (HyClone), and KHM-5M cells were cultured in RPMI-1640 (Gibco). All the aforementioned cell lines were maintained in medium supplemented with 10% fetal bovine serum (FBS) (ExcellBio, FSP500) and were authenticated via short tandem repeat (STR) assays. None of the cell lines showed any mycoplasma contamination.

### Cell transfection

Plasmids for GPX3 and JUN were purchased from Miaoling Bio (Wuhan, China) and transfected into CAL-62 and KHM-5M cells using Lipofectamine 3000 (Invitrogen; Waltham, MA, USA) according to the manufacturer's recommended protocol. The transfection was carried out in serum-free medium, and after 6 hrs, the medium was containing 10% FBS. Cells were then incubated for an additional 24-48 hrs before being harvested for subsequent experiments.

### MTT assay

Following transfection, 3 × 10³ TC cells were seeded into 96-well plates and cultured for five days. After 48 hrs, 20 μL of MTT solution was added to each well and incubated for 4 hrs. The resulting formazan crystals were dissolved in 200 μL of DMSO, and absorbance was measured using a spectrophotometer.

### Clonogenic assay

Approximately 1000 cells from each group were seeded into six-well plates. After 9-12 days of incubation, the cells were fixed and subsequently stained for analysis.

### Transwell assay

Cells from different experimental groups were seeded at a density of 2-10 × 10⁴ cells/200 μL into the upper chambers of a 24-well Transwell plate, which contained a polycarbonate membrane with appropriate pore size (Corning). After an incubation period of 16-24 hrs, the cells that had migrated through the pores to the lower surface of the membrane were fixed to maintain their structure. These cells were then stained to facilitate visualization and counting.

### Wound healing assay

Cells from different experimental groups were seeded into six-well plates and allowed to grow until they reached full confluence. Once the cell monolayer was 100% confluent, a straight scratch was carefully made through the cell layer using a pipette tip to simulate a wound. The cells were then cultivated in a medium without FBS for 0 or 16-20 hrs to prevent cell proliferation during the assay. The wound area was photographed at the beginning (0 hr) and after the incubation period (16-20 hrs) using a microscope to monitor the migration of cells into the scratched area.

### Western blot

The procedure followed established protocols. Cells were lysed on ice for 30 mins in a buffer containing RIPA, phosphatase inhibitors, and protease inhibitors. After high-speed centrifugation, the supernatant was gathered. Protein samples were separated by SDS-PAGE and then transferred onto PVDF membranes. The membranes were blocked using 5% skim milk in TBST buffer and subsequently incubated with primary and secondary antibodies. Protein detection was carried out using the Imaging System. The primary and secondary antibodies utilized are detailed in Supplementary Table 3, with GAPDH serving as the only internal control.

### Human tissue samples

The tissue microarray (TMA) consisted of 120 diagnosed thyroid cancer patients, including 58 cases of cancer and adjacent tissues, as well as 4 normal thyroid tissues. Immunohistochemical (IHC) staining was performed using an anti-GPX3 antibody. The analysis process did not take into account clinical outcomes, clinical characteristics, or pathological staging. Tissue section scanning and imaging were performed using a slide scanner. Positive scoring was conducted using the Densito quantification module in the Quant Center 2.3 analysis software: negative was scored as "0", weak positive as light yellow ("1"), moderate positive as light yellow ("2"), and strong positive as brown ("3"). The number of weak, moderate, and strong positive cells, as well as the total number of cells, were counted in the measured region. The positive cell percentage and the final positive score per point were calculated as follows: percentage: 1 < 25%, 2 = 25-50%, 3 = 50-75%, 4 > 75%. The staining signal of tumor cells was quantified using a 0 to 12 scoring system. The final score was obtained by multiplying the positivity level by the percentage of positive cells. Low and high expression were defined as scores < the average and ≥ the average, respectively.

### Statistical analysis

The data were analyzed using GraphPad Prism 9 and are presented as the mean ± standard deviation (SD). An unpaired two-tailed Student's t-test, one-way ANOVA, and two-way ANOVA were employed. Chi-square test was used to analyze the association between clinical characteristics and gene expression levels in high and low groups. The *p*-value < 0.05 was deemed statistically significant.

## Results

### Identification of DEGs

**Figure 2:**
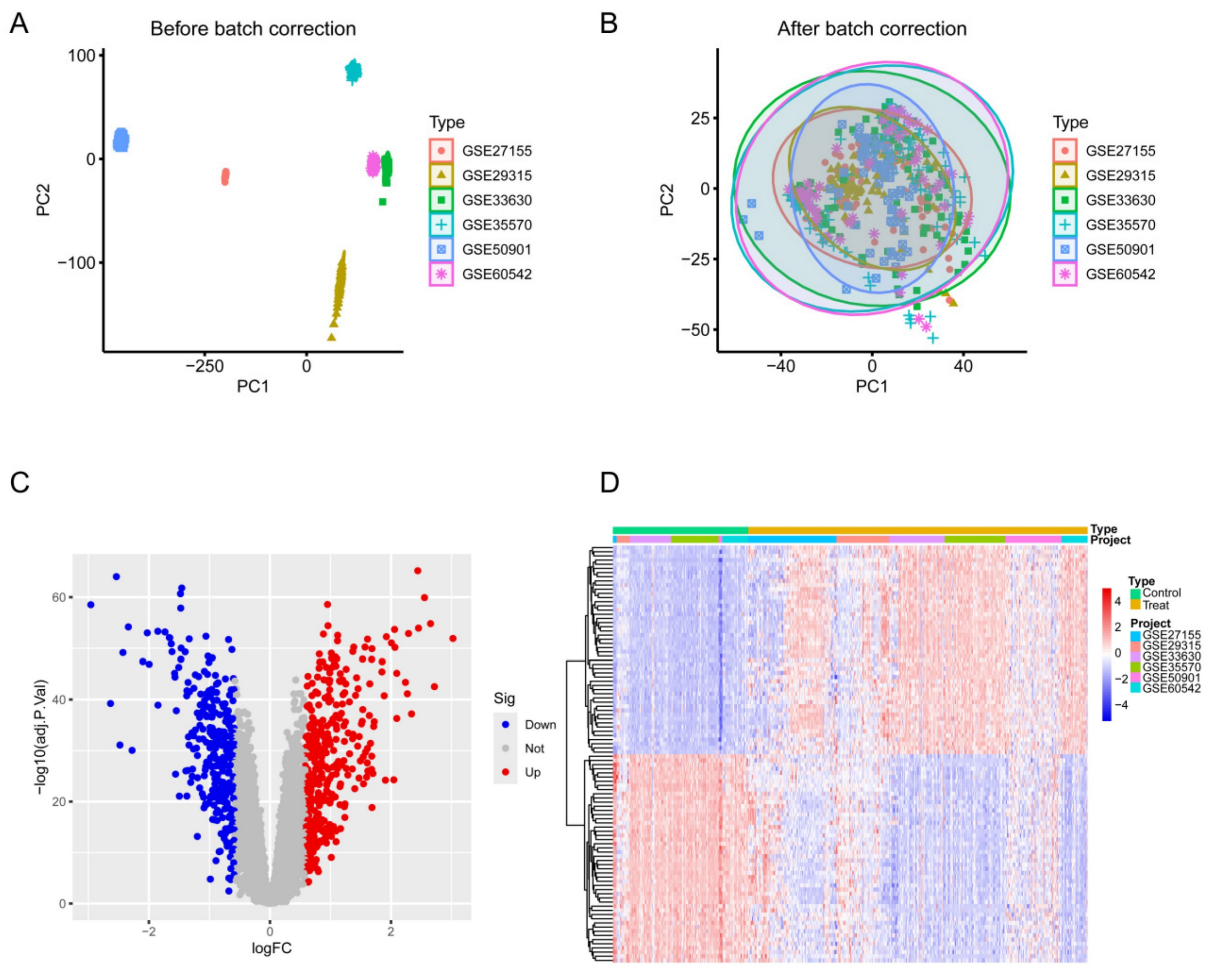
** Differential expression analysis of TC and normal thyroid tissue across six datasets.** (A) PCA before batch correction. (B) PCA after batch correction. (C) Volcano plot of differential expression analysis from the merged six datasets. (D) Heatmap of the top 50 DEGs in the merged datasets. Blue denotes downregulated genes, red signifies upregulated genes, and gray indicates genes with no significant change.

To identify key molecules potentially involved in the progression of TC, we conducted differential expression analysis on six datasets (GSE27155, GSE29315, GSE33630, GSE35570, GSE50901, GSE60542) obtained from the GEO database. The data were log_2_-transformed and batch effects were removed using the sva package. PCA demonstrated that the batch effects had been effectively mitigated in the corrected data (Figure 2A-B), and heatmaps (top 50; Figure 2C) and volcano plots (Figure 2D) were generated. This analysis identified 369 upregulated and 280 downregulated DEGs.

### Identification of TC-related module via WGCNA network

**Figure 3:**
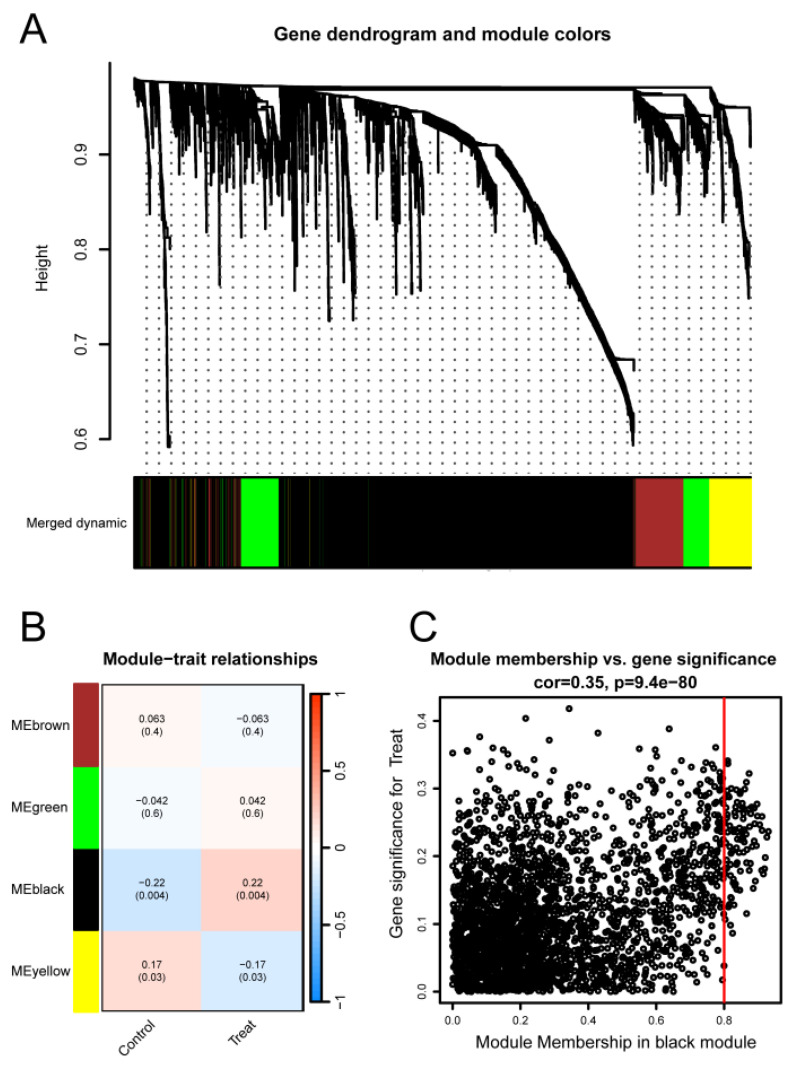
** Identification of TC-related gene modules using WGCNA in the GSE6339 Dataset.** (A) Dendrogram of all genes in the GSE6339 dataset, clustered based on the Topological Overlap Matrix (1-TOM). Each branch of the dendrogram represents a gene, and the co-expression modules are indicated by different colors. (B) Module-trait heatmap showing the correlation between clustered gene modules and TC in the GSE6339 dataset. Each module includes the corresponding correlation coefficient and *p*-value. (C) Scatter plot of the module eigengene from the black module, showing the strongest positive correlation with TC in the GSE6339 dataset.

To investigate the relationship between potential gene modules and TC, we performed WGCNA on the TC dataset (GSE6339) (Figure 3A). Through this analysis, we identified four distinct modules (Figure 3B). Subsequently, by examining the positive correlation coefficients, the black module showed the strongest correlation with cancer versus normal status (*p* = 0.004, R² = 0.22). Therefore, the black module was selected as the focus of this study. Further analysis revealed that the black module contained a total of 2739 genes (Figure 3C), with 1526 genes being upregulated and 1213 genes downregulated.

### Identification of risk genes associated with TC by MR analysis

**Figure 4:**
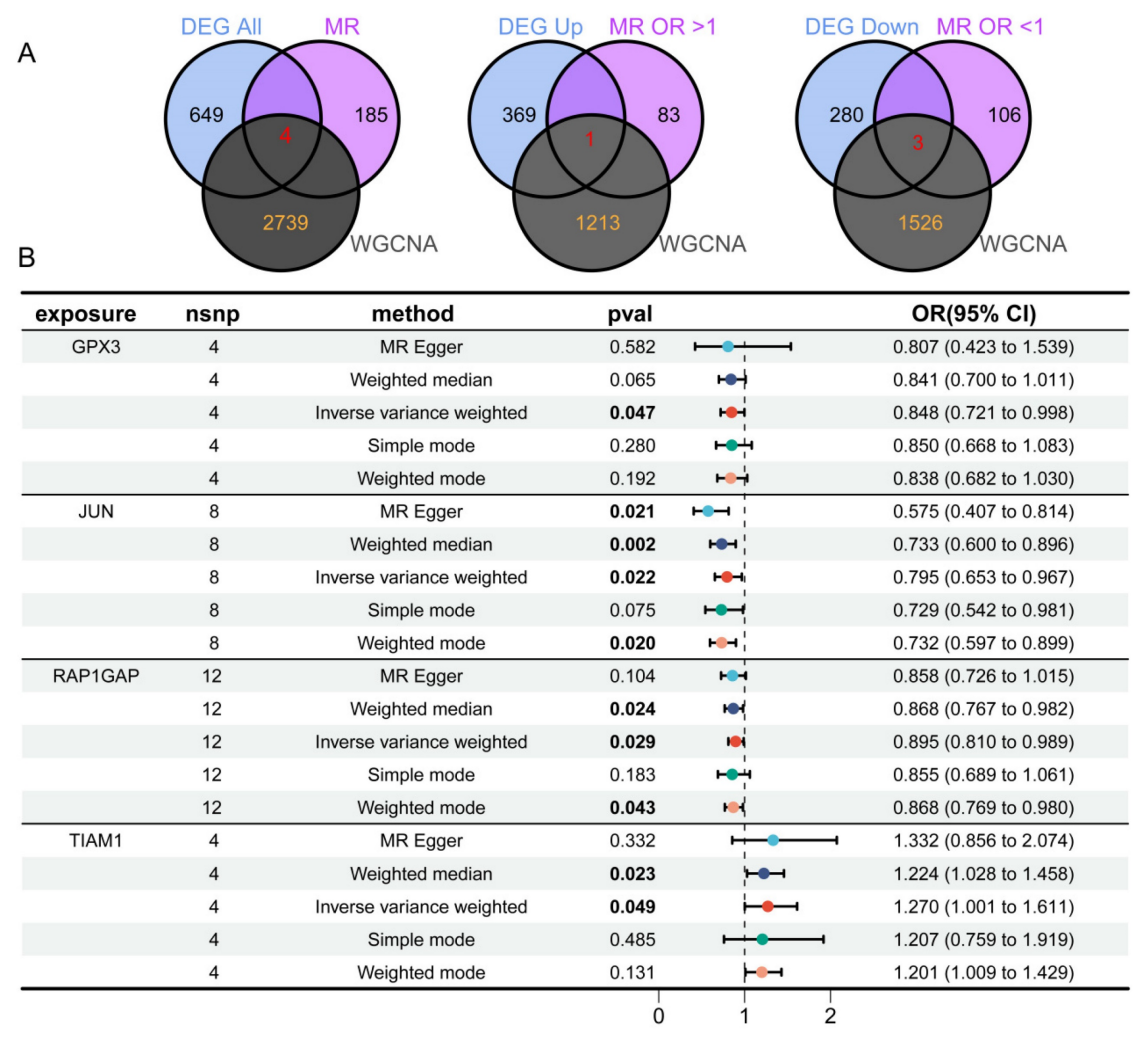
** Identification of four overlapping genes using three analytical methods.** (A) Venn diagram revealing four overlapping candidate genes, with one gene upregulated and three downregulated. (B) MR analysis shows that GPX3, JUN, and RAP1GAP offer protective effects against TC, while TIAM1 has an adverse influence on TC.

To identify eQTLs associated with TC, we downloaded the eQTL data and performed preprocessing steps to ensure data quality. After clumping, 5,428 genes were retained for MR analysis. Using the IVW method, we applied three selection criteria: concordant odds ratio (OR) values across five MR methods, heterogeneity *p-*value > 0.05, and other relevant parameters. Through this rigorous filtering process, we identified 189 genes with significant genetic associations with TC. Specifically, increased expression of 83 genes was significantly associated with higher TC risk, whereas increased expression of 106 genes was significantly associated with reduced TC risk. By integrating the results from the three aforementioned analyses (Figure 4A) and taking their intersection, we identified four key DEGs: TIAM1, RAP1GAP, JUN, and GPX3. Among these, three genes (RAP1GAP, JUN, GPX3) were downregulated, while TIAM1 was upregulated. This process culminated in the creation of forest plots summarizing the results from the five MR analysis methods (Figure 4B).

Next, we conducted a detailed MR analysis to visualize the causal effects between genetic variants associated with GPX3 (Supplementary Figure 1A,E), JUN (Supplementary Figure 1B,F), RAP1GAP (Supplementary Figure 1C,G), and TIAM1 (Supplementary Figure 1D,H) and TC. We assessed the causal relationships between GPX3, JUN, RAP1GAP, TIAM1, and TC. Using the IVW method, we identified significant associations between GPX3 and TC risk with an OR of 0.848 (95% CI 0.721-0.998, *p* = 0.047), JUN and TC risk with an OR of 0.795 (95% CI 0.653—0.967, *p* = 0.022), RAP1GAP and TC risk with an OR of 0.895 (95% CI 0.810—0.989, *p* = 0.029), and TIAM1 and TC risk with an OR of 1.270 (95% CI 1.001—1.611, *p* = 0.049). MR-Egger tests indicated no heterogeneity in the analyses for GPX3 (*p* = 0.989, Supplementary Figure 1I), JUN (*p* = 0.235, Supplementary Figure 1J), RAP1GAP (*p* = 0.470, Supplementary Figure 1K), and TIAM1 (*p* = 0.103, Supplementary Figure 1L). The MR-Egger regression intercepts, which assess pleiotropy, showed no evidence of pleiotropy for GPX3 (*p* = 0.890), JUN (*p* = 0.081), RAP1GAP (*p* = 0.550), and TIAM1 (*p* = 0.809), further supporting the robustness of our results. The leave-one-out sensitivity analysis revealed no significant impact of any single SNP on the outcomes, confirming the reliability of the causal effect estimates (Supplementary Figure 1M-P).

### GO and KEGG analysis

**Figure 5:**
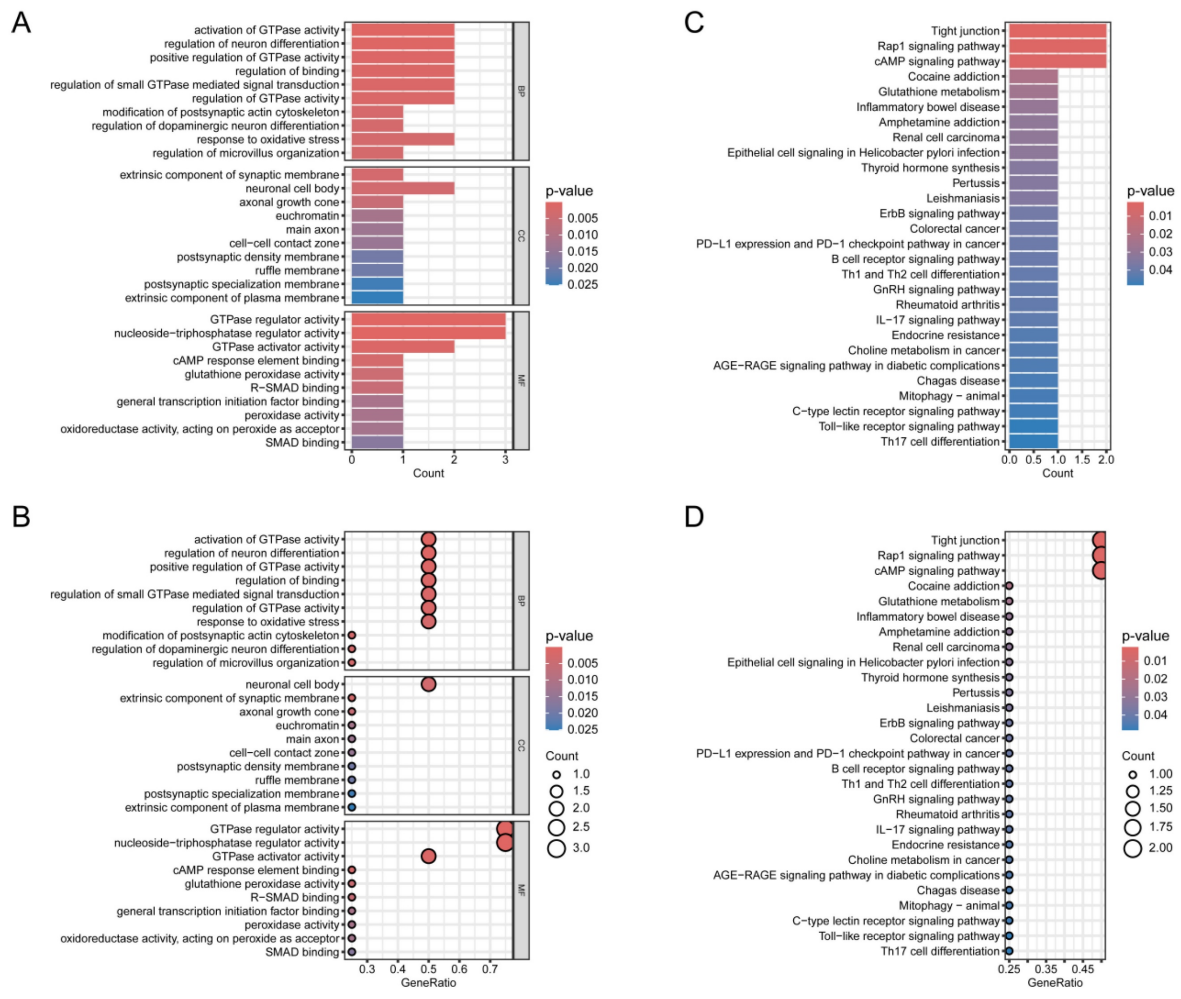
** Validation of candidate genes.** (A, B) Bubble plot and bar chart of the GO enrichment analysis for the candidate hub genes, respectively. (C, D) Bubble plot and bar chart of the KEGG pathway analysis for the candidate hub genes, respectively.

To further explore the hypothesized cellular functions and pathways associated with normal thyroid tissue and TC, GO and KEGG analyses were conducted on the four identified genes. First, GO and KEGG analysis was visualized through bar graphs (Figure 5A,B) and bubble charts (Figure 5C,D). The Biological Process (BP) analysis revealed enrichment in terms such as "regulation of modification of synaptic structure", "positive regulation of glial cell migration", and "regulation of microvillus organization". KEGG pathway analysis highlighted the top five enriched pathways: "Tight junction", "Sulfur metabolism", "Rap1 signaling pathway", "AMP signaling pathway", and "Selenocompound metabolism".

### Gene Set Enrichment Analysis (GSEA)

**Figure 6:**
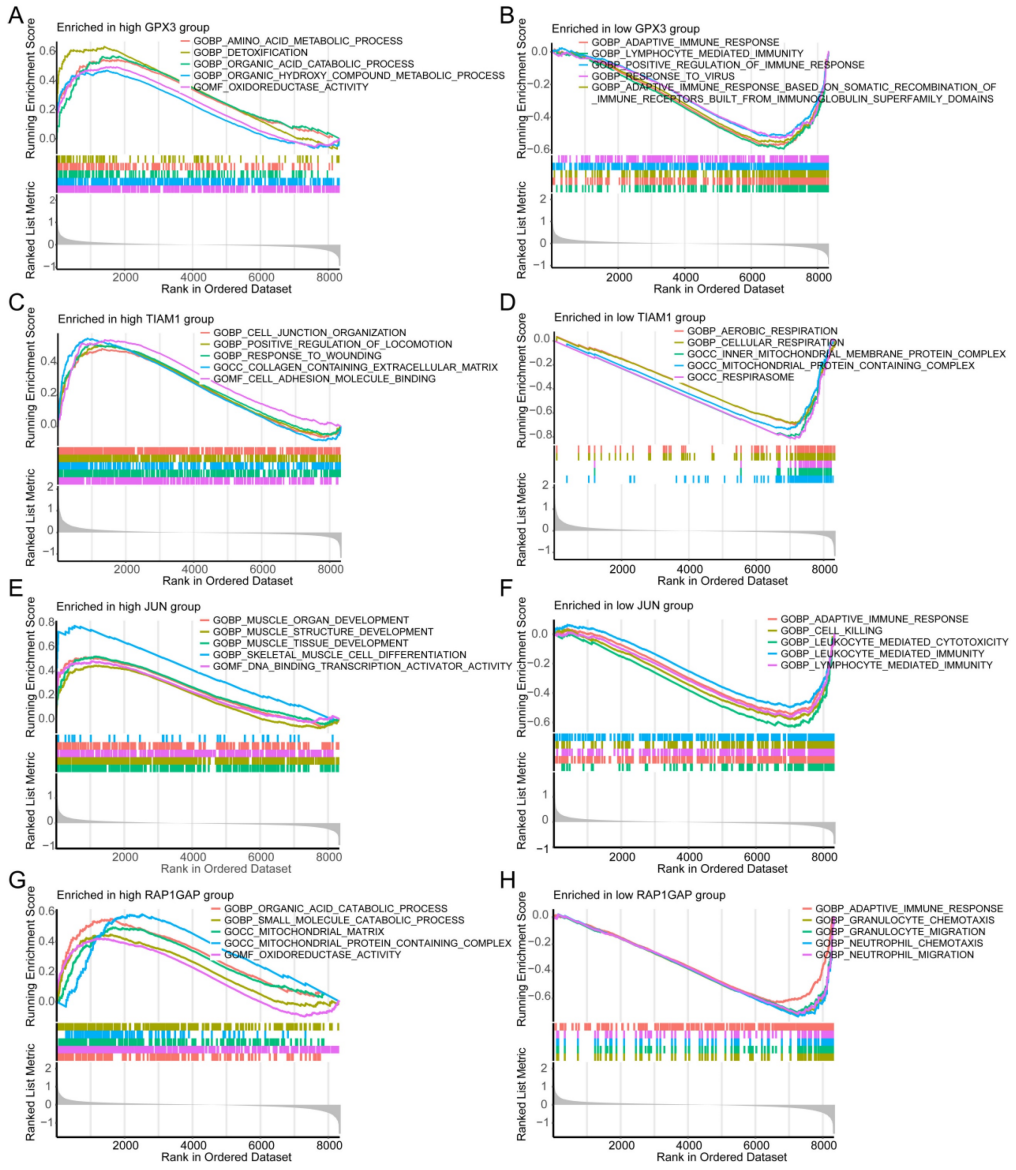
** Single-gene GSEA analysis for each of the four candidate genes using "c5.go.symbols.gmt" gene sets.** (A, B) Top five enriched GO terms for high- and low-risk groups in GPX3. (C, D) Top enriched GO terms for high- and low-risk groups in TIAM1. (E, F) Top five enriched GO terms for high- and low-risk groups in JUN. (G, H) Top five enriched GO terms for high- and low-risk groups in RAP1GAP.

GSEA was employed to uncover the distinct molecular functions associated with high and low risk of individual genes in TC. For high-risk GPX3 expression, the top enriched Gene Ontology Biological Processes were several metabolic processes, and "Oxidoreductase Activity" (Figure 6A). Conversely, low-risk GPX3 expression was significantly enriched in several immune response pathways (Figure 6B). For TIAM1, the high-risk group was significantly related to "cell junction organization", "positive regulation of locomotion", and "response to wounding" (Figure 6C). Conversely, the low-risk group showed enrichment in "aerobic respiration" and "cellular respiration" (Figure 6D). For JUN, the high-risk group was enriched in development processes of muscle organ, structure, and tissue (Figure 6E), while the low-risk group was enriched in "adaptive immune response", "cell killing", and "leukocyte-mediated immunity" (Figure 6F). Regarding RAP1GAP, the high-risk group demonstrated enrichment in catabolic proces of organic acid and small molecule (Figure 6G), while the low-risk group was enriched in "adaptive immune response", "granulocyte migration", and "neutrophil chemotaxis" (Figure 6H).

Additionally, we performed single-gene GSEA analysis based on KEGG pathways. The results indicated that the high-risk TIAM1 group was associated with processes such as "cell molecules (CAMs)", "complement and coagulation casc", "ECM-receptor interaction", and "focal adhesion" (Supplementary Figure 2A). In the low-risk group was linked to "fatty acid metabolism" and "oxidative phosphorylation" (Supplementary Figure 2B). For JUN, the high-risk group was enriched in the "Hedgehog signaling pathway", "selenium amino acid metabolism", "TGF-beta signaling pathway", and "tyrosine metabolism" (Supplementary Figure 2C), whereas the low-risk group was enriched in "allograft rejection" and "systemic lupus erythematosus" (Supplementary Figure 2D). The high-risk RAP1GAP group was associated with metabolism of butanoate, fatty acid, glycine, serine, and threonine (Supplementary Figure 2E). On the other hand, the low-risk group was enriched in pathways such as "allograft rejection" and "systemic lupus erythematosus" (Supplementary Figure 2F). For GPX3, the high-risk group showed significant enrichment in several amino acid metabolism and degradation pathways, including "arginine and proline metabolism", "butanoate metabolism", "Hedgehog signaling pathway", and "tyrosine metabolism" (Supplementary Figure 2G). In contrast, the low-risk group was associated with endocrine-related diseases, such as systemic lupus erythematosus and diabetes mellitus (Supplementary Figure 2H).

These findings indicate that each gene plays a distinct role in the biological pathways implicated in the development and progression of TC. For instance, high GPX3 expression is linked to metabolic and detoxification processes, suggesting a role in cellular redox balance and metabolism. In contrast, low GPX3 expression is associated with immune response pathways, highlighting its potential involvement in immune evasion or modulation within the tumor microenvironment. These insights provide a deeper understanding of the molecular mechanisms by which these genes may contribute to TC pathology and offer potential avenues for targeted therapeutic strategies.

### Immune infiltration analysis

**Figure 7:**
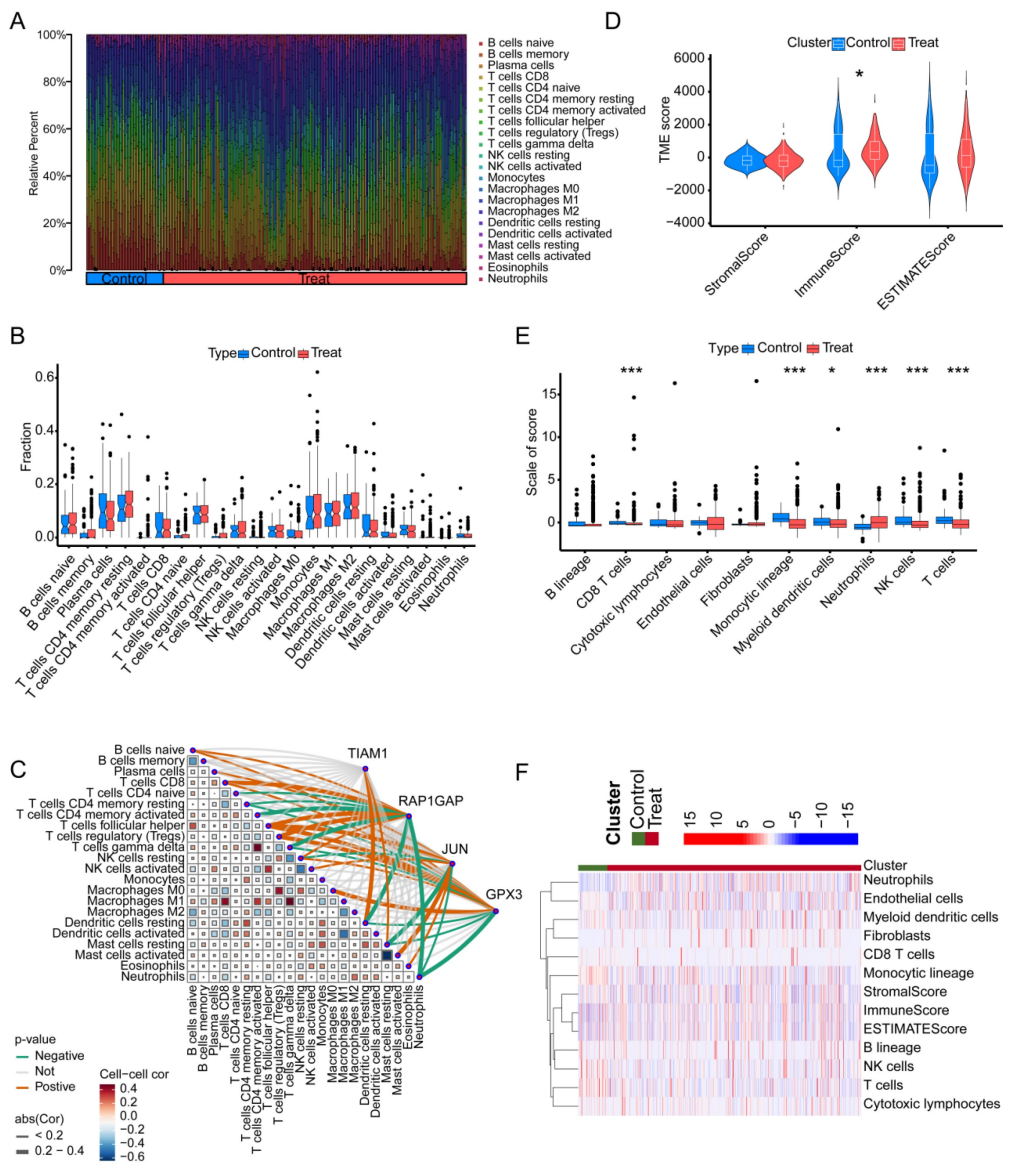
** The relationship between the four genes and immune infiltration in TC using different algorithms.** (A) Proportion of immune cell infiltration across different samples. (B) Differences in immune cell infiltration between TC tissues and normal tissues. (C) Correlation analysis between the four genes and 22 types of immune cells in TC. (D) Comparison of stromal score, immune score, and overall ESTIMATE score between TC and adjacent non-cancerous tissues. (E) Differences in 10 types of immune cells between TC and adjacent non-cancerous tissues, as determined by the MCPcounter algorithm. (F) Heatmap showing the levels of 10 immune cell types in each sample.

We employed the MCPcounter algorithm to assess immune infiltration in both tumor-adjacent tissues and TC across six GSE datasets. The analysis revealed that, compared to TC tissues, the adjacent normal tissues exhibited higher proportions of "Mast cells resting", "Mast cells activated", "Eosinophils", and "Neutrophils". Conversely, the proportions of "B cells naive", "B cells memory", and "Plasma cells" were lower in the adjacent tissues relative to TC (Figure 7A). Regrettably, under this algorithm, we did not observe any significant differences among the 22 types of immune cells between adjacent non-cancerous tissues and tumor tissues (Figure 7B). Further analysis integrating the expression of four key genes highlighted potential immune-related mechanisms through which these genes may contribute to TC progression. TIAM1 gene may influence TC progression by modulating the activity of "Dendritic cells resting" and "Mast cells resting". RAP1GAP gene appears to positively regulate TC progression by affecting "B cells naive", "T cells CD8", "T cells follicular helper", and "T cells regulatory". Additionally, it may negatively influence TC by affecting "T cells CD4 memory activated", "T cells gamma delta", "Dendritic cells resting", and "Neutrophils". JUN gene may contribute positively to TC progression through its impact on "B cells naive", "T cells follicular helper", and "Mast cells activated", while inversely affecting "Mast cells resting". GPX3 gene seems to play a role in TC by influencing "T cells follicular helper", "T cells regulatory", and "Macrophages M0". "Neutrophils" are the primary immune cells negatively regulated by GPX3 (Figure 7C). In summary, these findings suggest that the identified genes might exert their effects on TC by modulating specific immune cell populations, particularly "T cells regulatory" and "T cells follicular helper". To assess the immune cell infiltration patterns in TC patients, we applied the "estimate" function to score all adjacent normal and cancerous samples, obtaining StromalScore, ImmuneScore, and ESTIMATEScore. Notably, the ImmuneScore differed between adjacent normal tissues and cancerous tissues (Figure 7D). Further analysis using the "MCPcounter" package quantified the abundance of various immune cell types. The results revealed significant differences in the abundance of CD8 T cells, monocytic lineage cells, myeloid dendritic cells, neutrophils, NK cells, and T cells between adjacent normal and cancerous tissues. Specifically, with the exception of neutrophils, which were scored lower in adjacent normal tissues, the other cell types were likely more abundant in the adjacent normal tissues than in the cancerous tissues (Figure 7E). Subsequently, we utilized a heatmap to illustrate the differences in three scoring metrics and 10 types of cells between adjacent non-cancerous tissues and cancerous tissues (Figure 7F). This finding suggests that the depletion of immune cells, which play a crucial role in the anti-tumor response, may be an important factor in promoting cancer development and progression in TC.

### External dataset validation

**Figure 8:**
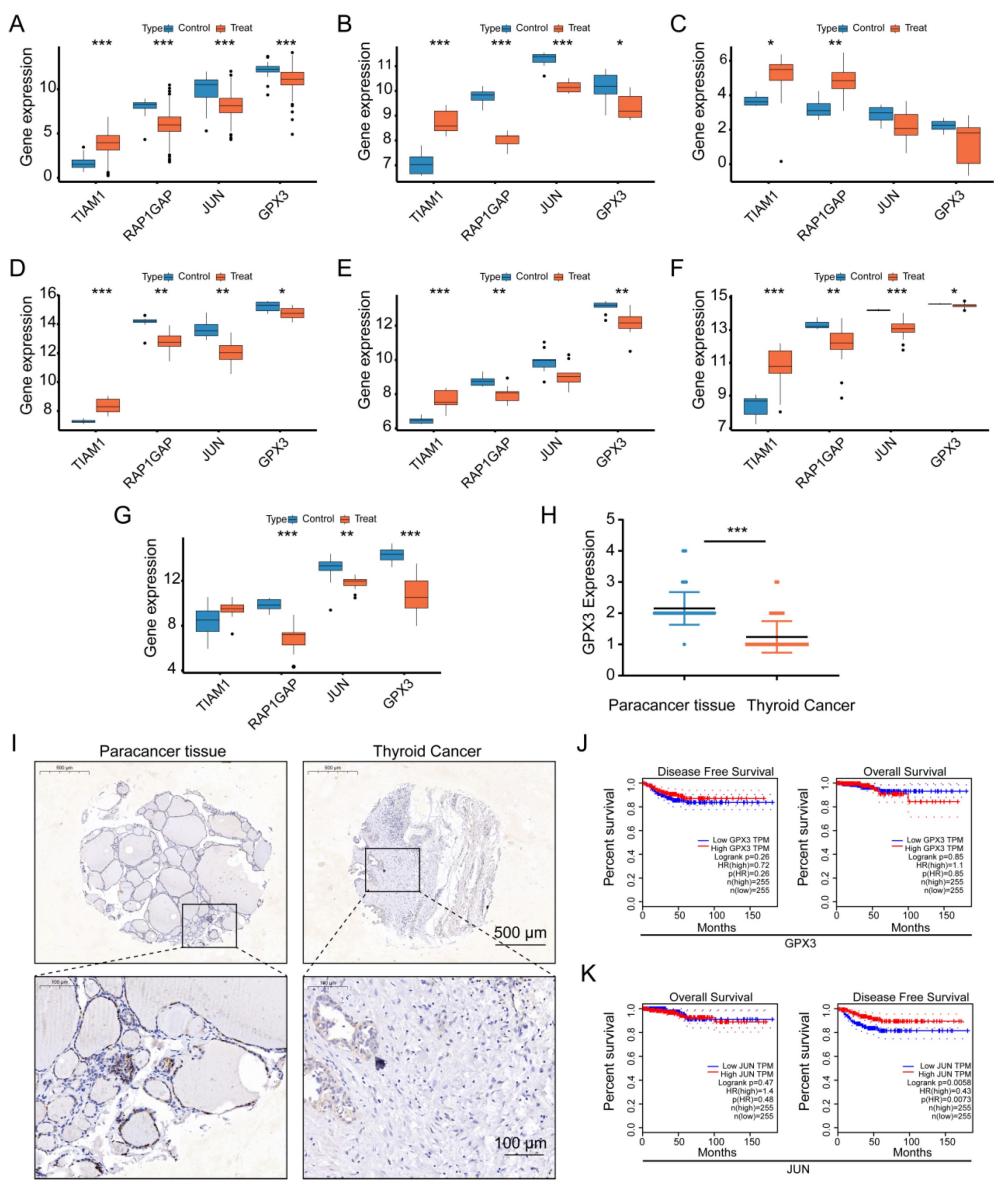
** External validation of the identified four genes across multiple datasets.** (A-G) Expression differences of GPX3, JUN, TIAM1, and RAP1GAP between normal tissues and TC tissues in the following datasets: TCGA (A), GSE3678 (B), GSE9115 (C), GSE129562 (D), GSE3467 (E), GSE104005 (F), and GSE65144 (G). (H) and (I) indicate that immunohistochemical staining images showing the expression levels of GPX3 in cancerous and adjacent normal tissues from 58 TC patients, with corresponding expression scores, scale bars: 0.5 mm, 100 μm (enlarged). (J) Survival analysis was performed to assess the relationship between GPX3 expression levels and overall survival (OS) as well as disease-free survival (DFS) in thyroid cancer prognosis. (K) Survival analysis was conducted to evaluate the association between JUN expression levels and OS and DFS in thyroid cancer prognosis.

To further validate the expression patterns of the four identified genes, we conducted an analysis using 6 GEO datasets (GSE3678, GSE9115, GSE129562, GSE3467, GSE104005, and GSE65144), as well as data from TCGA. The results showed consistent expression trends across these datasets (Figure 8A-G). For TIAM1, all seven datasets indicated higher expression in cancerous tissues compared to normal tissues, although one dataset did not reach statistical significance. In the case of RAP1GAP, all seven datasets consistently demonstrated lower expression in cancerous tissues, with all results being statistically significant. For JUN, the seven datasets showed lower expression in cancerous tissues, though two of the datasets did not achieve statistical significance. Similarly, GPX3 was found to have lower expression in cancerous tissues across all seven datasets, with one dataset lacking statistical significance. Overall, the seven datasets corroborated the differential expression of these four genes, consistently reflecting differences in expression levels across all datasets. This suggests that TIAM1 may function as an oncogene, while RAP1GAP, JUN, and GPX3 might act as tumor suppressors. However, further experimental validation is required to confirm these roles.

To further validate our findings, we performed immunohistochemical staining for GPX3 on 58 paired thyroid cancer (TC) and adjacent normal tissue samples, followed by scoring (Figure 8H-I). Compared to adjacent normal tissues, GPX3 expression was significantly lower in the clinical thyroid cancer samples. Additionally, survival analysis using TCGA prognostic data revealed that low expression of JUN was significantly associated with disease-free survival (DFS) in thyroid cancer patients (P = 0.0058) (Figure 8J-K). Although GPX3 expression did not reach statistical significance in relation to overall survival (OS) or DFS, a noticeable difference in OS was observed between patients with high and low GPX3 expression. Furthermore, by stratifying GPX3 and JUN into high and low expression groups based on the median, clinical characteristic analysis revealed a significant correlation between the expression levels of GPX3 and pathologic stage, stage T, and stage N. These findings suggest that GPX3 may serve as a potential predictor for the staging and lymph node metastasis of thyroid cancer.

### Cellular functional assay

Upon further literature review, we found that TIAM1 and RAP1GAP have already been reported as an oncogene and a tumor suppressor gene, respectively, in TC, with their roles validated through cellular biology experiments 35-37. Therefore, we focused our attention on the two key molecules, GPX3 and JUN , for functional validation in TC cells. Given their observed low expression in TC, we acquired plasmids for these genes and conducted ectopic overexpression experiments in TC cell lines. Initially, we confirmed successful overexpression of GPX3 and JUN in CAL-62 and KHM-5M cells (Supplementary Figure 4A-B).

**Figure 9:**
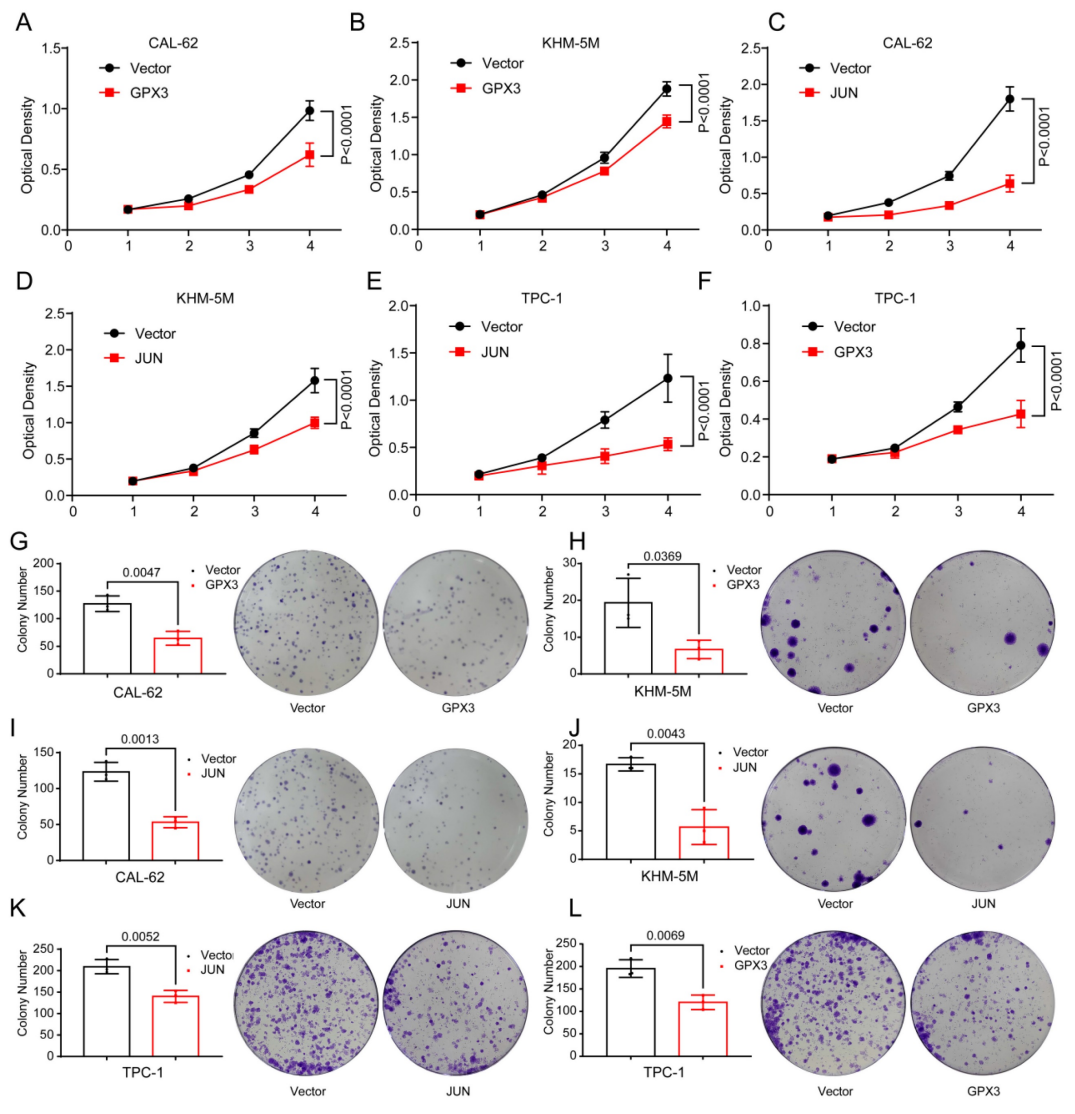
** Overexpression of GPX3 and JUN inhibits proliferation of TC cells.** MTT assay showing the proliferation of CAL-62 cells (A) and KHM-5M cells (B) after GPX3 overexpression. MTT assay showing the proliferation of CAL-62 cells (C) and KHM-5M cells (D) after JUN overexpression. MTT assay showing the proliferation of TPC-1 cells after JUN (E) and GPX3 (F) overexpression. Colony formation assay showing the proliferation of CAL-62 cells (G) and KHM-5M cells (H) after GPX3 overexpression. Colony formation assay showing the proliferation of CAL-62 cells (I) and KHM-5M cells (J) after JUN overexpression. Colony formation assay showing the proliferation of TPC-1 cells after JUN (I) and GPX3 (J) overexpression.

Subsequent functional assays revealed significant findings. In CAL-62 cells, overexpression of GPX3 resulted in markedly lower optical density (OD) values and fewer colony formations in the experimental groups compared to the control groups (Figure 9A,G), and the similar trend was observed in KHM-5M cells (Figure 9B,H). Overexpression of JUN resulted in markedly lower optical density (OD) values and fewer colony formations in CAL-62 cells compared to the control groups (Figure 9C,I), and the similar trend was observed in KHM-5M cells (Figure 9D,J). Overexpression of JUN resulted in markedly lower optical density (OD) values and fewer colony formations in TPC-1 cells compared to the control groups (Figure 9E,K), and the similar trend was observed in TPC-1 cells when GPX3 was overexpressed (Figure 9F,L).

**Figure 10:**
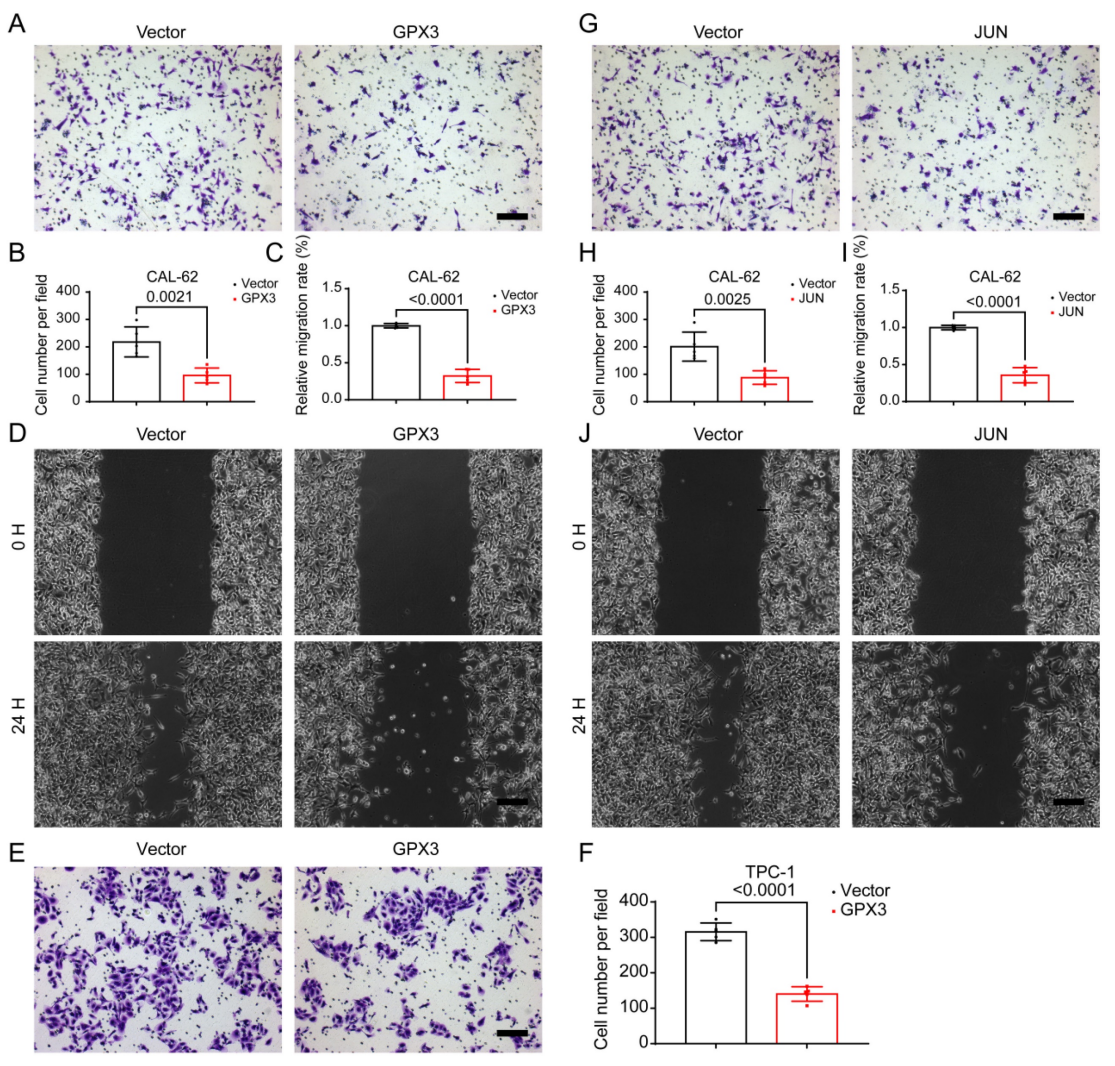
** Overexpression of GPX3 and JUN inhibits migration of TC cells.** Transwell assay showing the reduced ability of CAL-62 (A-B) cells to migrate through the chamber after GPX3 overexpression. Wound healing assay demonstrating the decreased migration ability of CAL-62 (C-D) cells after GPX3 overexpression. Transwell assay showing the reduced ability of TPC-1 (E-F) cells to migrate through the chamber after GPX3 overexpression. Transwell assay demonstrating the decreased migration ability of CAL-62 (G-H) cells after JUN overexpression. Wound healing assay showing the reduced ability of CAL-62 (I-J) cells to migrate through the chamber after JUN overexpression. All scale bars in this figure represent 200 μm.

Given that distant metastasis is also a hallmark of advanced-stage thyroid malignancies, we further investigated the migratory capabilities of TC cells. Further analysis using the Transwell assay demonstrated that the number of cells migrating through the membrane was significantly reduced in the GPX3 overexpression groups compared to controls in CAL-62 (Figure 10A-B) and KHM-5M (Supplementary Figure 3A-B) cells. Similarly, wound healing assays indicated that the area of migration after 24 hrs was substantially smaller in the GPX3 overexpression groups than in the control groups in CAL-62 (Figure 10C-D) and KHM-5M (Supplementary Figure 3C-D) cells, implying reduced migratory capabilities of the TC cells. Additionally, Transwell assay demonstrated that the number of cells migrating through the membrane was significantly reduced in the JUN overexpression groups compared to controls in CAL-62 (Figure 10G-H) and KHM-5M (Supplementary Figure 3G-H) cells. Similarly, wound healing assays indicated that the area of migration after 24 hrs was substantially smaller in the JUN overexpression groups than in the control groups in CAL-62 (Figure 10I-J) and KHM-5M (Supplementary Figure 3I-J) cells, implying reduced migratory capabilities of the TC cells. Transwell assay demonstrated that the number of cells migrating through the membrane was significantly reduced in the GPX3 (Figure 10E-F) and JUN (Supplementary Figure 3E-F) overexpression groups compared to controls in TPC-1 cells. We then conducted a preliminary investigation into the underlying mechanisms. Western blot analysis revealed that overexpression of GPX3 and JUN significantly increased the levels of mesenchymal markers such as N-cadherin, Vimentin, and Snail (Supplementary Figure 4A-B).

## Discussion

In recent years, a growing body of research has highlighted the critical roles of various molecules, such as BARF, in the initiation, progression, and metastasis of thyroid malignancies. While substantial evidence suggests that patients with differentiated TCs, such as papillary and follicular TCs, generally have favorable prognoses, anaplastic TC remains highly aggressive, with a notably poor prognosis. Although some studies have identified biomarkers associated with TC, the methodologies and sample types employed have often been limited, hindering deeper insights into the biological impact of these markers on TC. To comprehensively investigate the regulatory molecules influencing TC, we employed multiple bioinformatics approaches, identifying four genes closely associated with TC. Subsequent cellular biology experiments revealed that molecules such as GPX4 and JUN function as tumor suppressor genes, exerting significant effects on the progression and metastasis of TC.

Initially, we preprocessed and batch-corrected TC datasets from six different regions. Differential expression analysis was performed using the limma package, leading to the preliminary identification of several genes potentially associated with TC. We then selected the GSE6339 dataset for WGCNA to identify molecules significantly correlated with clinical features (cancerous vs. adjacent normal tissues). Finally, we analyzed gene-related expression quantitative trait loci (eQTL) in a large cohort of TC patients and healthy controls, uncovering genes linked to disease-related genetic variations. By integrating the results from these three analyses, we identified four candidate genes: TIAM1, RAP1GAP, GPX3, and JUN.

Next, we conducted functional and pathway analyses for these four genes. Initially, GO and KEGG analyses identified potential biological functions and pathways these genes might be involved in. Subsequently, single-gene GSEA revealed that GPX3 and JUN are primarily associated with metabolic and redox functions. This suggests that GPX3 and JUN may exert their anti-cancer effects in TC by regulating amino acid metabolism and influencing intracellular redox balance. Given the well-established link between TC progression and factors such as immune cells and the tumor microenvironment, we further performed immune cell composition analysis. The results indicated that these genes might exert their effects through the modulation of regulatory T cells and follicular helper T cells. Finally, we validated the expression levels of these four genes across seven GEO datasets and the TCGA dataset, confirming their differential expression.

We further reviewed existing studies on these four genes in TC, and the results aligned with our findings. TIAM1 has been identified as an oncogene in TC 35, a role that has been validated through cellular functional experiments. Similarly, RAP1GAP has been recognized as a tumor suppressor in TC and has been validated 36, 37,. Consequently, our subsequent research primarily focused on exploring GPX3 and JUN through cellular experiments. We conducted ectopic overexpression experiments to elevate the levels of GPX3 and JUN in cancer cells. First, we confirmed the successful expression of GPX3 and JUN via Western blot. Given that proliferative behavior is fundamental to the progression of malignant tumors, we used MTT and colony formation assays to demonstrate that upregulation of GPX3 and JUN significantly inhibits the proliferation of TC cells. Additionally, considering that thyroid malignancies often metastasize, we employed Transwell and wound healing assays, which revealed that upregulation of GPX3 and JUN significantly impairs the migratory capacity of TC cells.

The human GPX3 gene is located on chromosome 5q32, consisting of five exons spanning approximately 10 kb and encoding a 23 kDa protein that forms a homotetramer 5. GPX3 is a glutathione peroxidase protein that protect cells from oxidative damage 5. In recent years, it has been observed that promoter hypermethylation downregulates GPX3 expression in various human malignancies. However, the specific regulatory mechanisms remain unclear. GPX3 transcription has been shown to be regulated by peroxisome proliferator-activated receptor gamma (PPARγ). Additionally, the GPX3 promoter contains binding sites of hypoxia-inducible factor 1 (HIF-1) and specificity protein 1 (Sp1) 38. GPX3 typically exhibits differential expression patterns across various cancers, with promoter hypermethylation commonly observed in most tumors, including TC. For instance, hypermethylation of the GPX3 promoter is a common occurrence in human cancers 6. In gastric cancer, GPX3 hypermethylation has prognostic significance 39, 40. Conversely, in certain cancers, GPX3 methylation leads to reduced expression, in colorectal cancer, GPX3 promoter methylation serves as a predictor of sensitivity to platinum-based treatments 41. and serves as a prognostic marker in ovarian cancer 9. However, studies on myeloid leukemia [42, 43]and clear cell renal cell carcinoma 44, 45 have yielded inconsistent conclusions regarding GPX3 methylation and expression levels.

The JUN gene is located in the 1p32-p31 chromosomal region, a locus implicated in translocations and deletions associated with various human malignancies. JUN encodes a protein that directly binds to specific DNA sequences, playing a crucial role in regulating gene expression. Recent studies have shown that JUN facilitates the senescence-associated secretory phenotype and the recruitment of immune cells, contributing to the prevention of prostate cancer progression 11. Additionally, the AP-1/c-Jun~Fra-2 dimer has been implicated in c-Myc-driven hepatocarcinogenesis 13. In colorectal cancer (CRC) cells, JUN is involved in USP28 transcriptional activation 15. GPX3 suppresses the proliferation, migration, and invasion of pancreatic cancer cells by modulating the JNK/c-Jun signaling pathway and enhances their sensitivity to chemotherapy 46. In triple-negative breast cancer, the JNK/c-Jun/TNF-α signaling axis promotes PD-L1 expression 47. A study on breast cancer stem cells revealed that c-Jun/AXL stress signaling contributes to chromosomal instability tolerance during tumor progression 48. Moreover, targeting c-Jun can overcome tamoxifen resistance in estrogen receptor-positive breast cancer by inhibiting fatty acid oxidation 49. Targeting c-Jun is also considered a potential therapeutic strategy for bone metastasis in luminal breast cancer 50. As a transcription factor, c-Jun regulates GLUT1 during glycolysis and metastasis in breast cancer 51. Jun and MLL1 synergistically control H3K4me3 to influence colorectal cancer enhancer activity 12. In hepatocellular carcinoma, inhibition of the JNK/c-Jun-ATF2 pathway can overcome cisplatin resistance by downregulating galectin-1 52. Furthermore, c-Jun-mediated JMJD6 repair enhances radioresistance in hepatocellular carcinoma through the IL-4-activated ERK pathway 53. In gastric cancer, c-Jun directly regulates FOXK1, promoting cell proliferation, invasion, and metastasis 54.

In this study, we integrated multiple bioinformatics approaches to identify genes closely associated with TC, and further investigated their roles in the occurrence, progression, and metastasis of TC through clinical samples and cellular functional experiments. Although our study employed emerging methods such as MR and WGCNA to thoroughly evaluate these biomarkers, there are some limitations that should be acknowledged. First, the TC cases were collected from online databases, and the sample size was relatively small, which may introduce some bias. Despite correcting for batch effects, population differences across datasets may still exist. Additionally, the data used for the MR analysis were derived from European populations, necessitating further validation of our findings in other populations. In some parts of the analysis, we referred to genotype data from European populations used in Mendelian randomization studies. This dataset provides crucial support for genetic causal inference. However, as it is primarily based on European samples, the applicability of the results may vary across different populations due to factors such as genetic background, environmental influences, and cultural differences that could affect disease traits and gene expression. For example, Mendelian randomization analyses using European data may not fully represent other racial groups. We emphasize the need for more cross-population validation studies in the future, particularly in non-European populations. Nonetheless, our experiments and analyses have largely demonstrated the specific functions of GPX3 and JUN in TC, and their impact on TC, along with the underlying mechanisms, will be a key focus of our future research.

## Conclusion

Our study identified and validated the roles of GPX3 and JUN as tumor suppressors in the progression and metastasis of TC through various analytical methods. Specifically, we found that the expression of GPX3 and JUN is reduced in TC, and their overexpression significantly inhibits the proliferation and migration of TC cells.

## Supplementary Material

Supplementary figures and tables.
