# Supplementary material for: Identification of GPX3 and JUN as Tumor Suppressors in Thyroid Cancer through Integrated WGCNA and Mendelian Randomization
Source: J Cancer. 2025 Feb 18;16(6):1814–31. doi: 10.7150/jca.104687 (PMC11905407; doi:10.7150/jca.104687)
Supplement: Supplementary file 1 — Supplementary figures and tables. [file jcav16p1814s1.pdf]

Supplementary Information

Supplementary Figures

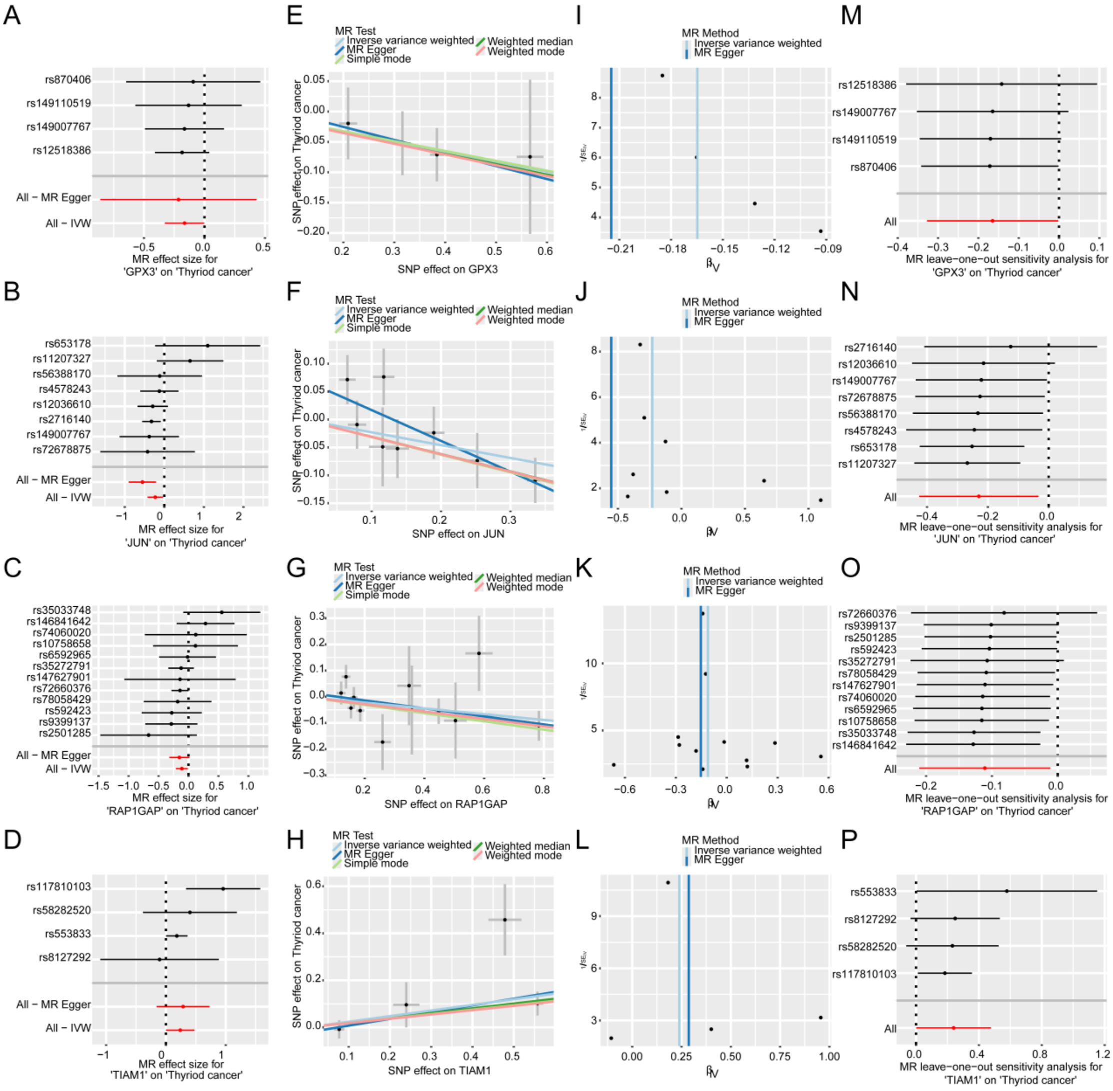

Supplementary Figure 1. MR analysis of the causal effect of four DEGs on TC risk. (A-D) Forest plots showing the causal effect of each SNP in GPX3, JUN, TIAM1, and RAP1GAP on TC risk. (E-H) Scatter plots depicting the causal effect of GPX3, JUN, TIAM1, and RAP1GAP on TC risk. (I-L) Funnel plots illustrating the overall heterogeneity of MR estimates for the effect of GPX3, JUN, TIAM1, and RAP1GAP on TC. (M-P) Leave-one-out plots visualizing the causal effect of GPX3, JUN, TIAM1, and RAP1GAP on TC risk when omitting one SNP at a time.

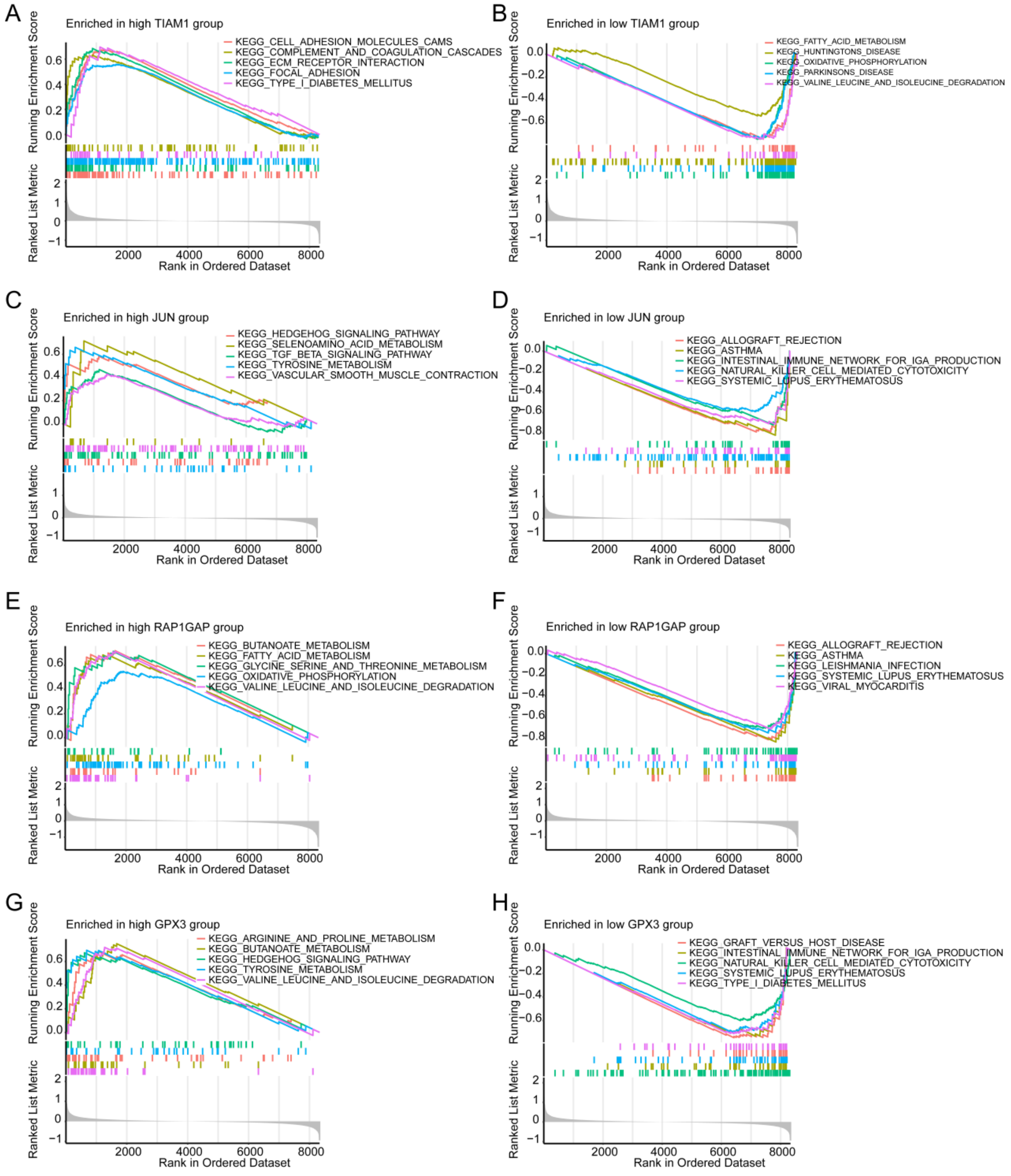

Supplementary Figure 2. Single-gene GSEA analysis for each of the four candidate genes using "c2.cp.kegg.Hs.symbols.gmt" gene sets. (A, B) Top five enriched KEGG pathways for high- and low-risk groups in TIAM1. (C, D) Top five enriched KEGG pathways for high- and low-risk groups in JUN. (E, F) Top five enriched KEGG pathways for high- and low-risk groups in RAP1GAP. (G, H) Top five enriched KEGG pathways for high- and low-risk groups in GPX3.

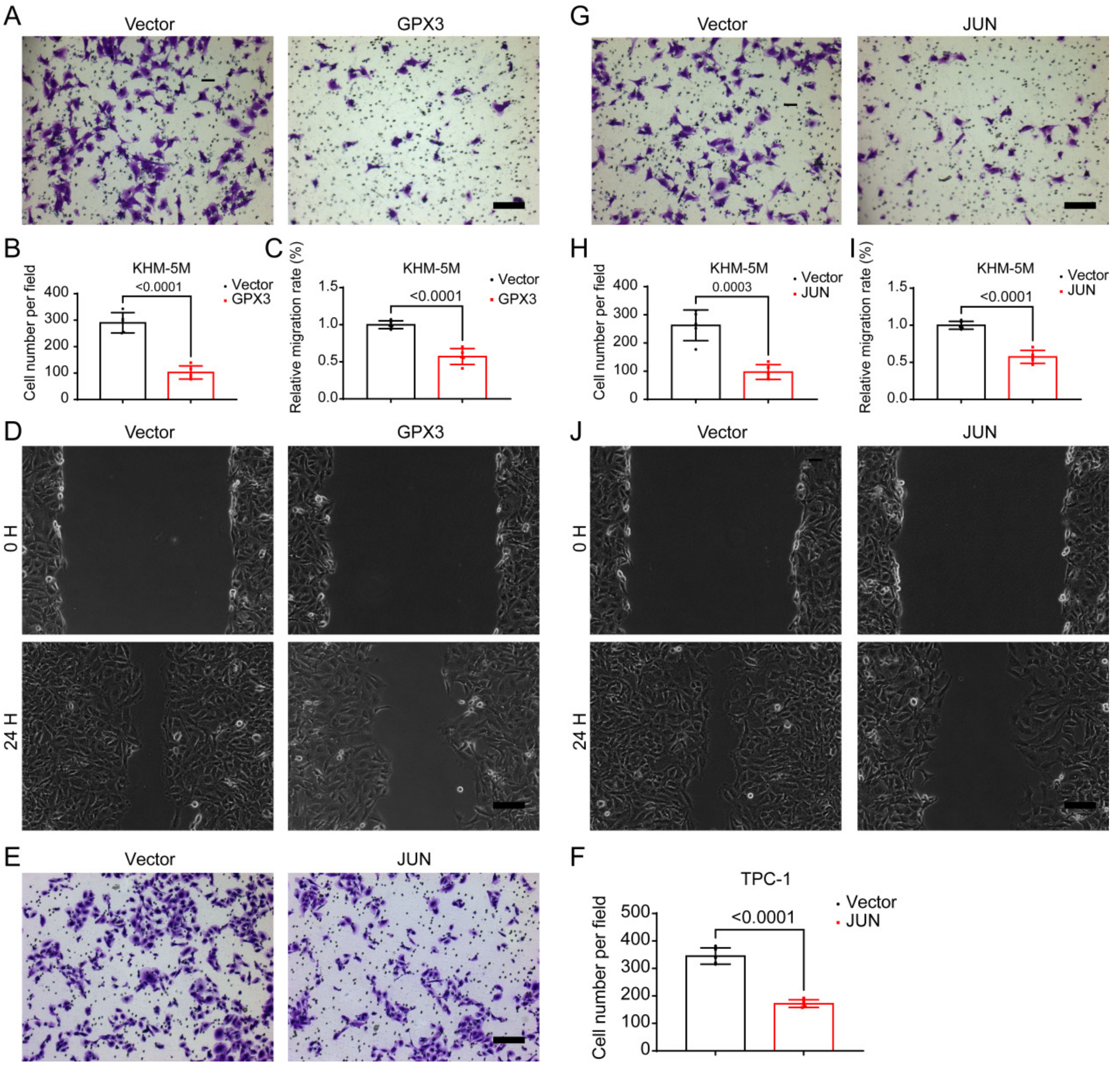

Supplementary Figure 3. **Overexpression of GPX3 and JUN inhibits migration of TC cells.** Transwell assay showing the reduced ability of KHM-5M (A-B) cells to migrate through the chamber after GPX3 overexpression. Wound healing assay demonstrating the decreased migration ability of KHM-5M (C-D) cells after GPX3 overexpression. Transwell assay showing the reduced ability of TPC-1 (E-F) cells to migrate through the chamber after JUN overexpression. Transwell assay demonstrating the decreased migration ability of KHM-5M (G-H) cells after JUN overexpression. Wound healing assay showing the reduced ability of KHM-5M (I-J) cells to migrate through the chamber after JUN overexpression. All scale bars in this figure represent 200 μm.

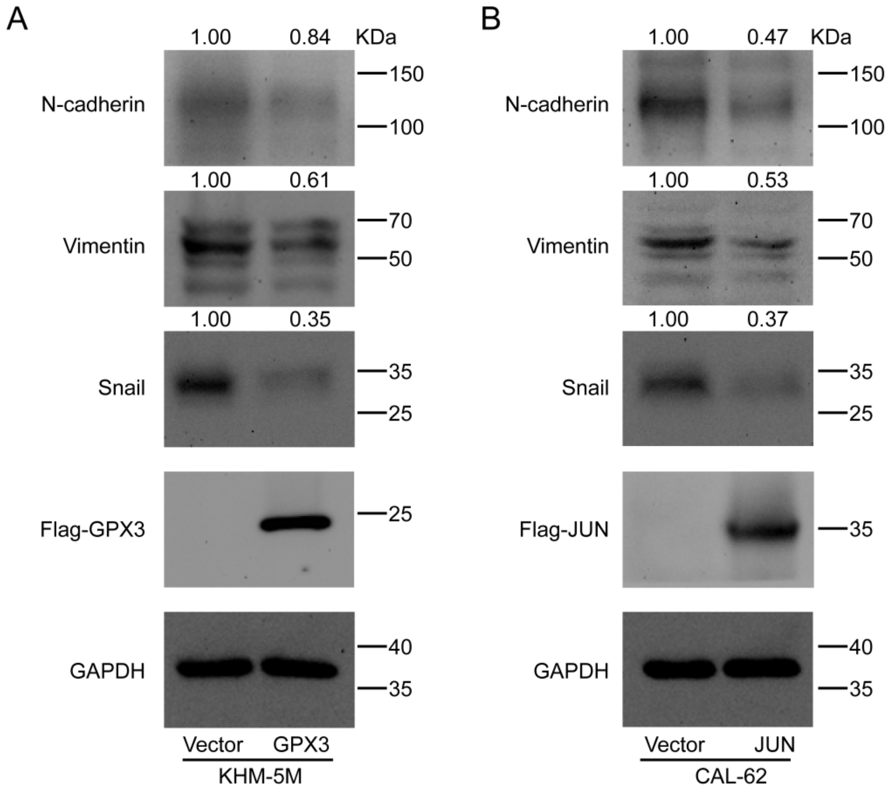

Supplementary Figure 4. Western blot analysis indicated expression in EMT-related proteins after overexpression of GPX3 in KHM-5M (A) and JUN in CAL-62 (B) cells. GAPDH was used as an internal control.

Supplementary Tables 1-3

Supplementary Table 1: Sample Information of all datasets from GEO and TCGA Databases

Group	Name	Platform	Control	treat(TC)	PMID
training set	GSE6339	GPL4512	73	93	17968324
	GSE27155	GPL96	4	95	16007166<br>16609000
	GSE29315	GPL8300	14	57	NA
	GSE33630	GPL570	45	60	22266856<br>22828610
	GSE35570	GPL570	51	65	26810418
	GSE50901	GPL13607	4	61	25867809<br>32425880
	GSE60542	GPL570	28	28	25965298
validation set	GSE3467	GPL570	9	9	16365291
	GSE3678	GPL570	7	7	30614796
	GSE9115	GPL9517	4	15	17981789
	GSE65144	GPL570	13	12	25675381
	GSE104005	GPL14951	6	30	31906302
	GSE129562	GPL10558	8	8	31498560
	TCGA-THCA	-	59	512	

Supplementary Table 2. Clinical characteristics of Thyroid Cancer.

	JUN				GPX3			
**Characteristics**	**Low**	**High**	**Chi-square**	**P**	**Low**	**High**	**Chi-square**	**P**
Age								
≤55	175	180	0.3084	0.5786	180	175	0.1751	0.6756
>55	78	72			73	77		
Gender								
male	71	65	0.3305	0.5654	62	74	1.515	0.2184
female	182	187			191	178		
race								
Asian	35	41	0.8166	0.6648	35	41	1.066	0.5870
White	205	196			202	199		
Other	13	15			16	12		
radiation								
NO	93	89	0.1138	0.7359	91	91	0.0011	0.9734
YES	160	163			162	161		
pharmaceutical								
NO	229	226	0.0978	0.7545	225	230	0.773	0.3793
YES	24	26			28	22		
Pathologic stage								
Stage I-II	160	176	2.47	0.116	156	180	5.411	**0.02**
Stage I-II	93	76			97	72		
Stage T								
T1-T2	156	153	0.0478	0.8274	144	165	3.895	**0.0484**
T3-T4，TX	97	99			109	87		
Stage M								
M0	142	140	0.1233	0.9402	149	133	4.151	0.1255
M1	4	5			2	7		
MX	107	107			102	112		
Stage N								
N0	110	120	0.9706	0.6155	102	128	11.02	**0.004**
N1	118	107			131	94		
NX	25	25			20	30		
Status								
Alive	245	244	0.0001	0.9936	248	241	2.348	0.1254
Dead	8	8			5	11		

Supplementary Table 3. Information for the primary antibodies used in this study.

Antibody	Supplier	Catalog	Application
N-cadherin	Proteintech	22018-1-AP	1:2000 for WB
Vimentin	CST	5741S	1:1000 for WB
Snail	CST	3879S	1:1000 for WB
GAPDH	Proteintech	60004-1-Ig	1:10000 for WB
GPX3	Abbkine	Cat. ABP58707	1:1000 WB
GPX3	Abbkine	Cat. ABP58707	1:500 IHC
Flag-Tag	Sigma	F1804	1:1000 for WB
